# Usability evaluation of the Computer-Based Health Evaluation System (CHES) eDiary for patients with faecal incontinence: a pilot study

**DOI:** 10.1186/s12911-022-01818-5

**Published:** 2022-03-28

**Authors:** Jens Lehmann, Isabel Schreyer, David Riedl, Michael Tschuggnall, Johannes M. Giesinger, Marjiana Ninkovic, Marcus Huth, Irmgard Kronberger, Gerhard Rumpold, Bernhard Holzner

**Affiliations:** 1 Department of Psychiatry, Psychotherapy, Psychosomatics and Medical Psychology, University Hospital of Psychiatry II, Medical University of Innsbruck, Anichstraße 35, 6020 Innsbruck, Austria; 2 Evaluation Software Development, Innsbruck, Austria; 3 Department of Visceral, Transplant and Thoracic Surgery, Medical University of Innsbruck, Innsbruck, Austria; 4 University Hospital of Psychiatry I, Medical University of Innsbruck, Innsbruck, Austria

**Keywords:** Ecological momentary assessment, Mobile applications, Patient reported outcome measures, Testing and evaluation, Monitoring and surveillance, Telemedicine and telehealth, Mobile computing and communication, Urology

## Abstract

**Background:**

Faecal incontinence (FI) is prevalent in 15–20% of elderly individuals and is frequently monitored in clinical trials and practice. Bowel diaries are the most common way to document FI, but, in clinical practice, are mainly used as paper-based versions. Electronic diaries (eDiaries) offer many potential benefits over paper-based diaries. The aim of this study was to develop and test an eDiary to document FI.

**Methods:**

We migrated a paper FI diary to an eDiary app based on the Computer-based Health Evaluation System (CHES). To assess usability, we conducted functionality and usability tests at two time points in a sample of patients with FI. In the first assessment, the eDiary functionalities were tested, patients completed the System Usability Scale (SUS, range 0–100) and compared the paper diary with the eDiary. We set a threshold for minimum acceptable average usability at 70 points. Patients were then instructed to use the eDiary for 2 days at home and contacted to report on their usage and completed the SUS a second time.

**Results:**

We recruited a sample of N = 14 patients to use the eDiary. All patients were able to use all functionalities of the eDiary and only a few patients with lower technological literacy or access to devices (n = 3) needed initial assistance. The mean usability rating given at the first time point was high with 88 points (SD 18, 95% CI 78.2–96.8) and most patients (n = 10) reported they would prefer the eDiary over the paper-based version. Nine patients (n = 9) participated in the follow-up assessment and the mean SUS rating at the second time point was 97 points (SD 7, 95% CI 92.8–100).

**Conclusion:**

The eDiary showed excellent usability scores for the assessment of FI at both assessments. Generally, patients preferred the eDiary over the paper-based version. We recommend the eDiary for usage with patients who own and use a smartphone and discuss potential solutions for patients with lower technological literacy or access.

**Supplementary Information:**

The online version contains supplementary material available at 10.1186/s12911-022-01818-5.

## Background

Faecal incontinence (FI) is defined as an unintentional and recurrent loss of faecal material for the duration of at least 1 month in adult individuals [1]. FI is prevalent in 15–20% of elderly individuals [2, 3]. Since physicians seldom screen for FI and patients often are ashamed to report such symptoms, FI is largely overlooked and underreported [3], although it is a physically and psycho-socially debilitating condition with a devastating impact on patients’ quality of life (QOL) [4]. A common way to assess FI is using bowel diaries. They facilitate the continued assessment of key outcomes in the diagnosis and management and are commonly used in clinical trials for FI [5, 6]. Bowel diaries enable a detailed assessment of characteristics of bowel symptoms and frequency of incontinence episodes in real time, also called ecological momentary assessment (EMA, [7]). Therefore, compared to other methods such as traditional questionnaire studies, diaries are ‘closer to life’ and are less susceptible to recall bias [8].

Today, paper diaries are still the most common form of bowel diaries in clinical trials and practice, despite being cumbersome to carry and complete in real time and offering only questionable accuracy [9]. Paper diaries also suffer from the disadvantage of possible and untraceable retrospective data entry. A study by Stone et al. [9], showed that, besides lower compliance for using the paper versus electronic diary (eDiary), participants using paper diaries often retrospectively enter data for multiple events (‘hoarding’), and that this occurs less when using eDiaries. Subsequent studies revealed that lower compliance of paper diaries also depends on the mode of administration (and may be overcome with some effort) and that psychometric equivalence of paper and eDiaries may be reached [10]. However, the problem of possible retrospective data entry still stands. Electronic assessment methods offer the possibility of limiting or monitoring the extent of retrospective data entry.

There are now several studies that show that the use of eDiaries can improve compliance of using a diary, while also reducing patient burden [11–13]. For example, eDiaries are successfully utilised in clinical trials, where they are used to monitor symptoms or medication intake and where they show high acceptability and compliance [14]. Electronic diaries are also frequently used in mood research [15, 16], in addiction research [17], and for symptom assessment such as pain [11, 18, 19]. For patients with FI, first evidence suggests that patients prefer electronic phone-application-based administration over paper diaries as they were considered easy to use and also had the benefit of producing high-quality data [20]. However, in the trial by Zyczynski et al. [20], the app was only tested in women from a specific trial population. There is still a lack of software that is developed in cooperation with patients and feasible for usage by patients with FI. This is important because, ultimately, the involvement of end users increases the likelihood of the software being used [21]. While there are a number of freely available applications to document bowel movements and FI, they suffer from a lack of quality and often cannot adequately document episodes of FI [22]. Even more importantly, secure storage and automated sharing of patient data with healthcare professionals or clinical study teams are not supported. Therefore, such applications may offer some benefits for the individual patient, but suffer from inadequate documentation of FI and a lack of integration into clinical practice or trials.

### Aim

In this study, we developed an eDiary for the documentation of FI by patients. The components of the eDiary were constructed to correspond to a paper–pencil diary used in clinical trials [23, 24]. We based the evaluation of the eDiary on processes outlined in the International Society for Pharmacoeconomics and Outcomes Research (ISPOR) guidelines for the migration of paper-based patient-reported outcome measures to an electronic format and measurement equivalence between the versions [25]. Consequently, the aims of the study were:

- to evaluate the usability of the eDiary for patients with FI,
- to migrate the paper-based version of the diary to an eDiary and compare the two versions, and
- to collect feedback to improve the eDiary for use in future clinical trials.

## Methods

### eDiary software

**Fig. 1:**
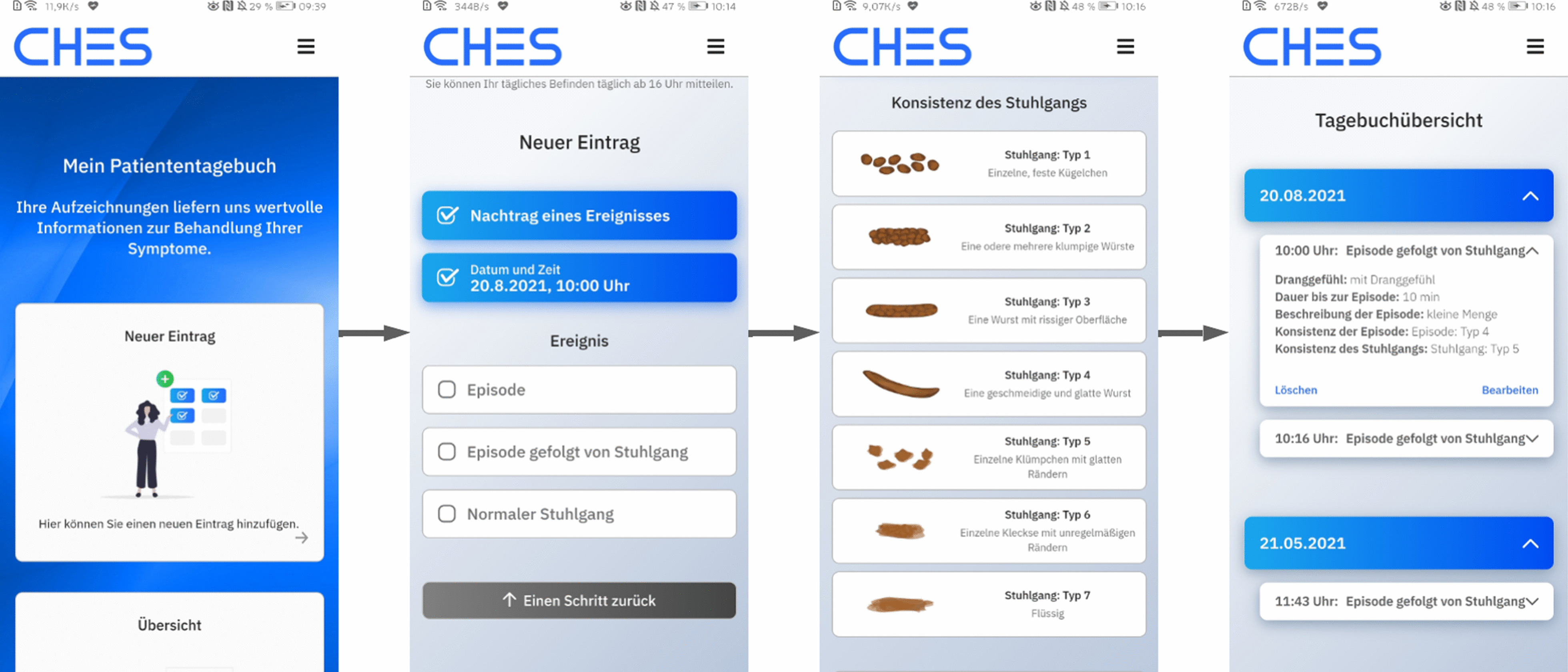
eDiary user interface in German language. From left to right: home area, entering a new event, choosing stool consistency from the Bristol Stool Chart, overview of diary entries

The eDiary was developed to enable the comprehensive patient-reported assessment of FI and bowel movements in clinical routine and for clinical trials. We used the Computer-based Health Evaluation System (CHES) [26–28] as a software basis to develop an iOS and Android app that can be downloaded on most mobile devices. Alternatively, the app can also be accessed via any web browser (no installation needed), although this way it does not offer all of the same features (e.g., no push notifications). Figure 1 shows the user interface of the main features. The eDiary contains the following functionalities (see Additional file 1, which provides original German language screenshots of all features of the eDiary):

- Logging in using the personal login data
- Entering events of FI or regular bowel movements
- Assessing open and closed questions on the nature of FI events, including the Bristol Stool Chart [29]
- Reviewing previously entered events of FI
- Modifying previously entered events of FI
- Deleting previously entered events of FI

The eDiary was accompanied by an information sheet explaining its use and giving individualised login details.

As the eDiary is based on CHES, it also features a healthcare professional interface (‘CHES.main’), where data are stored and can be inspected. This allows clinicians or study personnel to monitor patients’ data and possibly use the data in clinical practice [26]. However, this interface was not part of the current study, as we focused on the development of the patient eDiary which was primarily developed for the documentation of FI. The review of entered data and clinical use are possible, but were beyond the scope of our study.

### Development approach

We adapted our development process to the ISPOR guidelines for the validation of electronic systems to collect patient-reported outcome data [30]. Relying on systematic and constant communication with clinical trial experts, we created a tailored web app with respect to system design and eDiary expectations. To make the app available on iOS and Android devices, we further embedded the web app into native mobile applications, which were made available for download from the Google Play Store and the Apple App Store, respectively. To ensure code quality throughout the whole development phase, we relied on internal developer code reviews, semi-/automatic unit testing, and system testing conducted by a separate quality assurance group.

### Procedure

According to the ISPOR guidance for measurement equivalence [25], cognitive debriefing and usability testing should be applied if only minor changes were necessary to migrate from a paper-based version to an electronic one. We conducted usability assessments at two consecutive time points in a pilot population of patients with FI. The first assessment was conducted at the hospital after recruitment, the second one was conducted as a telephone follow-up assessment. All experimental protocols were approved by an ethics committee of the Medical University of Innsbruck (app. number 1377/2020) and were conducted in accordance with the Declaration of Helsinki.

### Sample

We recruited patients with a diagnosis of faecal incontinence. Patients were sampled to represent different age groups (below 65 years of age vs. equal to or above 65 years of age) and different patient-reported internet usage frequencies (infrequent, i.e., a maximum of 3 days a week vs. each day). We also oversampled lower education levels as lower education may be associated with somewhat lower computer literacy. See the “Data analysis” section for the calculation of the sample size.

Inclusion criteria were (1) a diagnosis of FI, (2) German language fluency, (3) basic computer literacy and internet access at home, and (4) providing informed consent.

### Initial assessment at the hospital

Patients were recruited at the University Hospital of Innsbruck, Department of Surgery and asked to participate in the study. After patients had provided informed consent, they were introduced to the eDiary by a study investigator and handed the information sheet and login and usage instructions for the eDiary. The study investigator then helped patients install the eDiary on their smartphone (if they had one). Patients were instructed to read the material and to complete tasks covering all software features of the eDiary at the study site, i.e.:

- to navigate to the app,
- to log in,
- to enter a (hypothetical) defecation event,
- to review all entered events (overview),
- to change a previously entered event (e.g., enter additional data),
- to delete a previously entered event, and
- to give a total assessment of how many complaints FI had caused them on that day.

If the patient did not have an internet-ready device with them (e.g., a smartphone), a smartphone was provided for initial completion. During the completion of the tasks, patients were asked to ‘think out loud’, i.e., to voice their thoughts while navigating the software. Concurrent think-aloud is an often-used technique for testing usability of software and gives insight into participants’ thoughts and emotions while using the software [31, 32]. The study investigator instructed participants to describe their thinking processes and ideas or thoughts while completing the tasks in the software and noted comments voiced by patients on a notepad. If the patient failed to complete a task, the investigator noted the reason for failure and gave more instructions on how to complete the task. This process was repeated until the patient successfully completed all tasks. Afterwards, patients were asked to complete the paper–pencil version of the System Usability Scale (SUS; see below for more information). If a patient had difficulty with reading the questions, the study assistant provided them verbally.

Finally, patients were also presented with the paper–pencil version of the diary. They were asked to compare the eDiary they had just tested and asked to judge differences and benefits/drawbacks of the versions using a short ad-hoc questionnaire.

### Follow-up assessment at home

Patients were then handed the information sheet/user login data and asked to complete the diary at home the following day using the login information. If they did not experience an FI event or bowel movement, they were instructed to enter hypothetical events. Following completion of the at-home assessment, patients were contacted by phone 2 days later to complete a cognitive debriefing interview regarding the usability of the eDiary at home. The interview addressed the following questions:

- Were you able to enter an event? (if not, why not?)
- Would you prefer the eDiary to a paper–pencil alternative?

If patients had been unable to complete the at-home assessment task, the interviewer recorded the reason (e.g., technical problems, motivational problems) and provided help or additional instructions if necessary. Patients were then given an additional day to complete the task and called up the following day to be interviewed again. As after the initial interview, patients were asked to complete the SUS, which was administered verbally.

**Fig. 2:**
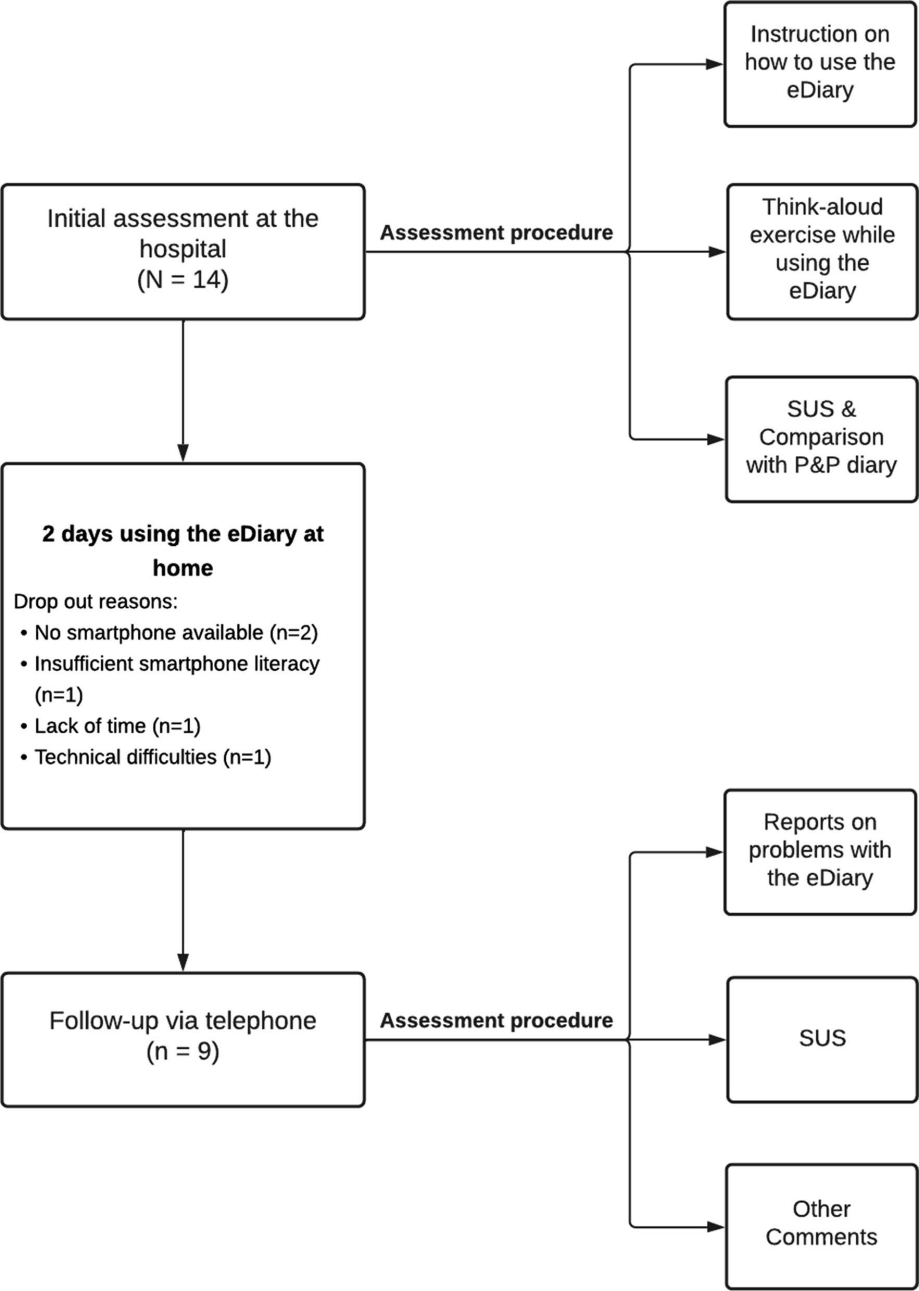
Study procedure flowchart. *SUS* System Usability Scale, *P&P* paper and pencil

The steps of this research are presented in Fig. 2.

### System Usability Scale (SUS)

The System Usability Scale (SUS) [33] is a 10-item questionnaire using a 5-point Likert scale evaluating users’ perceived system satisfaction, including two sub-scales of usability and learnability. The SUS is technology independent, frequently used and has received the maximum score in a recent quality appraisal of measures assessing system usability [34]. Over the last 30 years, the SUS has proven to be valid and reliable, but it is not diagnostic. It does not measure specific factors that contribute to the feasibility of a product, but allows comparisons between different systems and detects differences in smaller sample sizes than other questionnaires. Scores range from 0 to 100, but do not present percentiles (i.e., a score of 60 does not indicate a usability of 60%). Instead, a score of 70 points or higher is often used as initial threshold for acceptable usability, which was the overall mean of over 200 combined studies on different systems or products [35].

### Questionnaire to compare the paper-based diary and eDiary

A short questionnaire was constructed to compare the two versions. It contained three questions: (1) Do you see any differences between the two versions? (yes/no); (2) Which version of the diary would you find easier to use? (paper-based/eDiary/both are equally easy); (3) Which version of the diary would you choose to document your FI over a longer period of time? (paper-based/eDiary/either).

### Data analysis

We ran a power analysis to determine the mean SUS value needed to reliably show usability above the threshold for good usability of 70 points [35]. A sample size of N = 14 allows us to show this with 80% power and alpha = 0.05 (one-sided) if the observed group mean is 0.7 standard deviations (SD) above the threshold. Based on Sauro & Lewis [36] we assume a SUS SD of 12.5 points; thus, an observed group mean of 79 points would show good usability. Moreover, the sample size was deemed adequate as usability tests aim to find large effects or problems with the software and instrument. Such large effects can already be found with sufficient accuracy in smaller samples of 5 to 8 users [36].

Patient characteristics and patient completion (or completion failure) of the tasks in the eDiary are given as descriptive statistics. Patient comments regarding usability of the eDiary were transcribed and are presented separately. Quantitative data were collected with the SUS and are reported as mean and SD with a 95% confidence interval together with the percentage of patients exceeding the cut-off of 70 points indicating good acceptability [33].

## Results

### First assessment (at the hospital)

We approached 14 patients for participation in the study in the Department of Surgery at the Medical University of Innsbruck. All patients agreed to participate and provided informed consent. Patients’ sociodemographic data and information on their internet usage are reported in Table 1 (for the supplementary table also containing relationship status, occupation, and living situation, see Additional file 1). On average, patients were 67.4 years old (SD 10.7) and 92% used the internet at least once a week. Patients’ self-reported bowel problems are reported in Additional file 1. The data from the study are available (anonymized) in Additional file 2. Two patients did not own a smartphone and were provided with a smartphone for usage at the hospital by the study investigator.

**Table 1:** Patient characteristics

Characteristics	N = 14
Sex N (%)	
Male	3 (21)
Female	11 (79)
Age	
Mean	67.4
SD	10.7
Education N (%)	
Compulsory school graduation (apprenticeship)	10 (72)
Matura (further education)	3 (21)
University degree	1 (7)
Internet usage N (%)	
Confident in internet knowledge—yes	9 (64)
Confident in internet knowledge—no	6 (36)
Devices used to access the internet (multiple answers possible) N (%)	
Desktop PC	3 (21)
Laptop	2 (14)
Tablet	3 (21)
Smartphone	10 (71)
Frequency of internet usage N (%)	
Once per month	1 (8)
One to three times per week	2 (17)
Once per day	3 (25)
Multiple times a day	6 (50)
Missing^a^	2

^a^Missing data were not included in the calculation of percentages

The results for the individual tasks in the eDiary (e.g., logging in, entering an event) are reported in Table 2. Three patients (21%) had problems navigating to the eDiary or when logging in. These patients were provided personal assistance to complete those tasks. However, once logged in, all patients were able to enter, review, modify, or delete an event in the eDiary. After the first seven patients observed in the study, no new errors arose, which indicates saturation of problem identification.

**Table 2:** Patients’ performance in the eDiary

Evaluation or task	At hospital (N = 14)	At home (N = 9)
Information reported comprehensively in eDiary		
Yes	14 (100)	n/a
No	0	n/a
Able to navigate to the eDiary on own smartphone		
Yes	11 (79)	9 (100)
No, unable to type on smartphone	1 (7)	0
No, font too small	1 (7)	0
No, other technical problems	1 (7)	0
Able to log in to the eDiary		
Yes	11 (21)	9 (100)
No, unable to type on smartphone	1 (7)	0
No, font too small	1 (7)	0
No, other technical problems	1 (7)	0
Able to enter an event		
Yes	14 (100)	9 (100)
No	0	0
Able to review an existing event		
Yes	14 (100)	8 (89)
No	0	1 (11)
Able to modify an existing event		
Yes	14 (100)	n/a
No	0	n/a
Able to delete an existing event		
Yes	14 (100)	5 (56)
No, did not try to do this	0	4 (44)
Able to navigate forward and backwards in the questions		
Yes	n/a	8 (89)
No, did not try to do this	n/a	1 (11)

n/a not applicable, as this question was not assessed for this time point

### Second assessment (follow-up at home)

Nine patients participated in the follow-up assessments from home. Reasons for not being able to participate in the follow-up assessments were not owning a smartphone (n = 2), insufficient smartphone literacy (e.g., only using the smartphone for calls) (n = 1), a lack of time (n = 1), and technical difficulties (n = 1).

At the second assessment, patients were able to complete almost all tasks in the eDiary (see Table 2); for some functionalities (reviewing or deleting an event), some patients reported not having tried to do this at home. There was only one case where a patient was not able to review an existing event (which was discovered to be a user error, since the patient did not remember where to click). There were no software bugs or problems reported that hindered patients in using any of the eDiary functionalities.

### System usability evaluation at the hospital and at home

Patients reported high usability of the eDiary. At the hospital, 12 patients (84%) gave a SUS rating of at least 70 points, which was the predefined threshold for acceptable usability. The mean usability rating given at the hospital was 87.5 points (SD 17.8, 95% CI 78.2–96.8). Of the patients who could be contacted for follow-up, all (N = 9, 100%) gave a rating of at least 70 points (mean rating 97.2 points, SD 6.7, 95% CI 92.8–100). It has to be noted that patients that participated only at the hospital, but not at the follow-up had a lower mean SUS score than those who did (mean rating 75.2 versus 98.2 points).

**Fig. 3:**
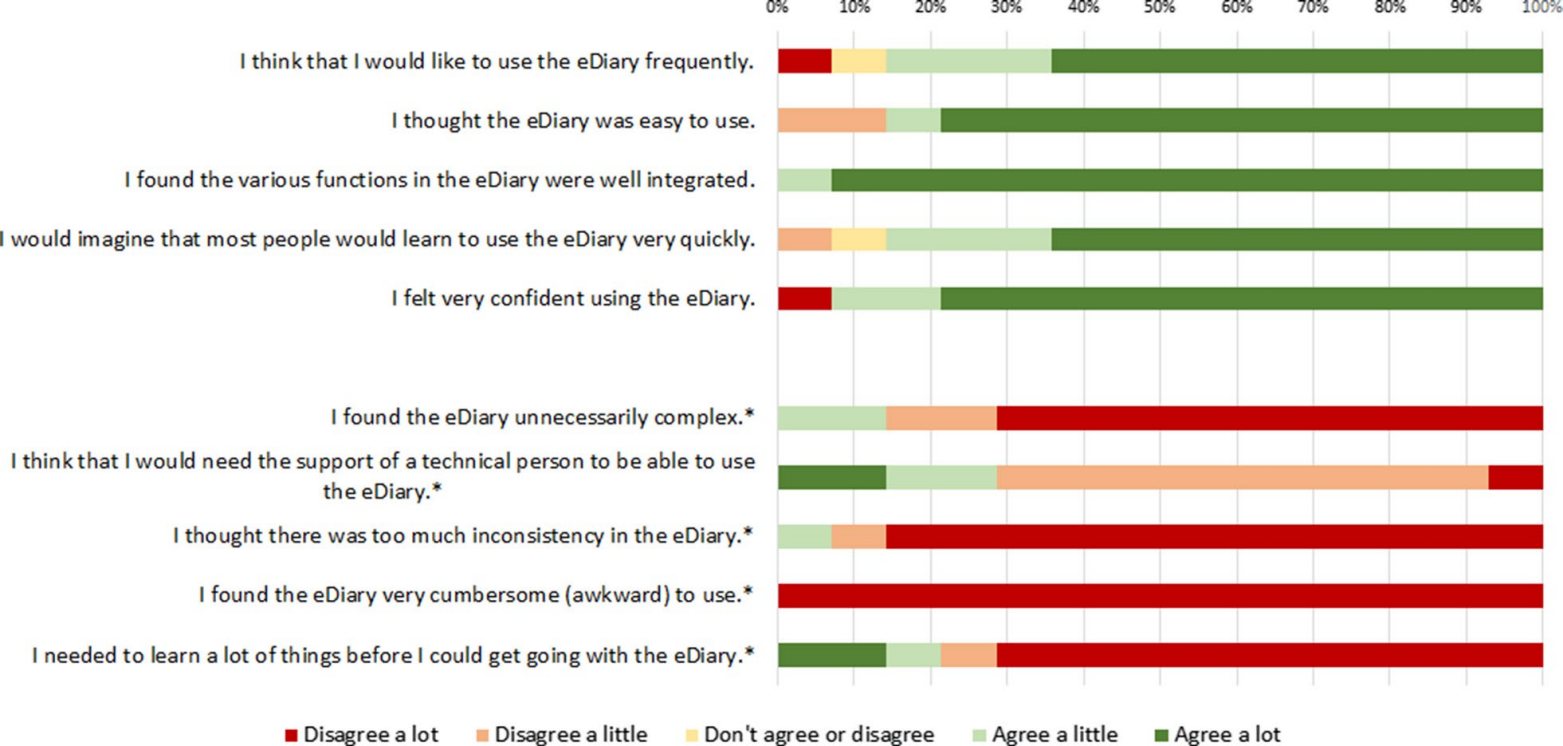
System Usability Scale ratings given by patients at the hospital. *Note*: * for items with asterisk, agreement indicates negative usability

**Fig. 4:**
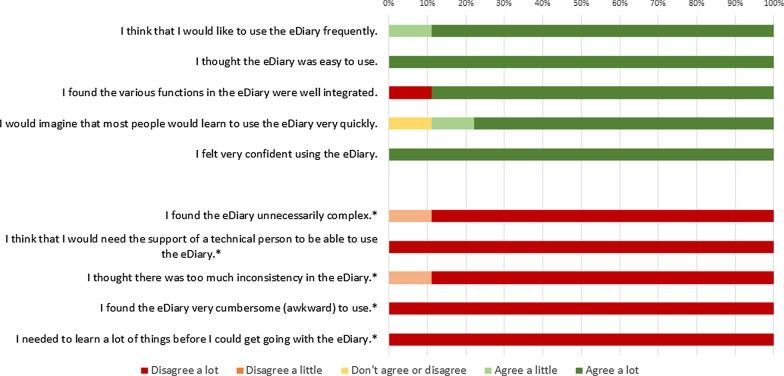
System Usability Scale ratings given by patients at home. *Note*: * for items with asterisk, agreement indicates negative usability

Figures 3 and 4 show the distribution of answers for the SUS rating at the hospital and at home. At the hospital, 10 patients (71%) reported an ‘excellent’ usability score, which is between 90 and 100 points. At home, 8 patients (89%) reported an ‘excellent’ usability score. The SUS items with the highest agreement at the hospital were “I found the various functions of the eDiary well-integrated” (N = 13, 93% “Agree a lot”) and “I thought the eDiary was easy to use” (N = 11, 79% “Agree a lot”). The SUS items with the highest agreement at home were “I felt confident using the eDiary” and “I felt the eDiary was easy to use” (both with N = 9, 100% “Agree a lot”).

### Comparing the eDiary and paper–pencil version

When asked to compare the eDiary with the paper–pencil version, 10 patients (71%) said that they would prefer to use the eDiary rather than the paper–pencil version for documenting their FI over a longer time period. Three patients (21%) said they would prefer the paper–pencil version and one patient (7%) said they did not prefer any version. In general, 11 patients (79%) did not see major differences between the two versions. Three patients reported differences. Paraphrased, one patient said the paper–pencil version gives too much information at once; one said that the paper–pencil version is too much to read at once and that this was better done in the eDiary; and one said that the paper–pencil format was more convenient and displayed the stool consistency pictures better. However, the same patient said that when documenting FI at night, the eDiary would probably be better. Regarding the ease of use, 10 patients (71%) said they found the eDiary easier to use, while 3 patients (21%) found the paper–pencil version easier to use, and 1 patient (7%) did not prefer any version.

### General comments

During the first assessment at the hospital, eight patients offered general comments regarding the software. Paraphrased, one patient said that they would need a contact person should they use the eDiary for a longer period in case they needed help. Two patients commented that the arrows for setting time intervals (see Additional file 1) in the eDiary were too small and it was hard to set them to the correct time. Another patient commented that the ‘sense of urgency’ could be present for less than 5 min (in the eDiary this was the minimum duration possible).

Patients also provided comments regarding the items of the diary itself. One patient commented on the term ‘episode’, which they found was not fitting. One patient commented on the stool consistency pictures, saying they were decent for documentation, but that sometimes they did not know or see the consistency of their stools. A similar comment was given by a second patient. The patient also suggested additional questions. One patient proposed adding a question on the size of the episode/defecation.

## Discussion

In this study, we evaluated a newly developed eDiary for the documentation of FI. Patients reported high satisfaction and high usability ratings for the eDiary. The majority of patients reported preferring the electronic version over a paper–pencil version. Patients suggested a few minor improvements.

### Patients’ understanding and use of the eDiary

Some patients required in-person assistance to download the app from the app-store and while entering log-in data as these patients were not used to typing on the smartphone. Once the eDiary had been installed, all patients were able to use it as intended, i.e., to document, review, modify or delete an event of FI. This shows that the eDiary can effectively replace a paper–pencil version while offering the same (or even extended) functionality. Similarly, in a review by Burton et al., it was stated that the accuracy of electronic diaries was found to be sufficient for research purposes since the generated data has shown to be valid for symptom research [11]. A study performed by Quinn et al. used electronic and paper–pencil diaries to document overactive bladder with or without incontinence. The eDiary proved to be more reliable and efficient compared to the paper-based version [37].

The majority of patients in our study were able to complete all documentation steps in the eDiary. Problems with the ease of use only arose in cases where the patients did not own a smartphone and were therefore untrained in their usage. In those cases, patients showed difficulties with the use of the eDiary and required help from the study investigator. As one would expect, an eDiary is only feasible for patients who own a smartphone and use it at least occasionally. A lack of access to special devices and knowledge has shown to be the most common barrier to technology use in healthcare. [38, 39] For patients who do not own a smartphone, the paper–pencil version is therefore recommended for the documentation of FI.

### Preferences of paper–pencil versus eDiary

Most patients reported that they would prefer the eDiary over the paper–pencil version for documenting FI. This conforms to findings from various studies comparing eDiaries with paper–pencil versions [18, 37, 40]. Benefits of the eDiary in our study concerned improved comprehensibility and display of information in the eDiary. The patients mentioned most frequently that the eDiary was preferred over the paper–pencil version because too much information was presented at once on the paper diary. The display of information step by step made filling out the eDiary easier. In general, the eDiary was rated as more favourable and easier to use than the paper–pencil version.

### Usability evaluation

In both evaluations, patients reported high usability for the eDiary. At the hospital and at home, more than 70% of patients reported ‘excellent’ usability and only two patients reported a usability below the set threshold of 70 points. Similar findings were reported by Zyczynski et al. [20]; high SUS scores were reached when comparing their smartphone diary app to a paper pencil version. Our present study and newly developed eDiary was able to increase the usability scores even further. This finding reflects the aforementioned preferences for the eDiary over the paper–pencil version and the low number of difficulties with the eDiary. Although our eDiary app was primarily developed to assess FI in a study context, the use of the app for FI documentation and self-reporting can be beneficial for patients living in rural or remote settings with limited access to healthcare. If used in clinical practice, it may allow patients to communicate their health status and FI documentation with their healthcare professionals and thereby support care management.

### Future improvements and implications for use

Even though usability ratings were high, areas for improvements were identified based on patients answers and comments. One frequently appearing topic is the size of text and features in apps and also in our eDiary. Patients remarked the small size of a pointer to select a time window hindering correct selection. Such elements should be increased in size, even if at the drawback of being able to fit less information into a single screen. A second improvement concerns the documentation of the sense of urgency in the eDiary. Patients mentioned that times below 5 min should be possible, as this was set as the minimum time window.

Finally, an implication for use of the eDiary, especially for patients not using their smartphones much, would be to provide assistance during the installation process. Some patients required help when logging in for the first time, as they had trouble correctly typing a username and password on their own (afterwards, the app logged them in automatically). Other patients had trouble initially finding the app on their smartphone. Both of which could be solved with the help of the study coordinator, but it also implies that, occasionally some assistance might be needed when distributing the app in future studies.

### Limitations

This study was conducted as a usability test and did not test for full psychometric equivalence between the paper-based diary and the electronic version. However, according to the ISPOR guidance, this can be considered sufficient as no major changes between the versions were made [25]. Another potential limitation is the small sample size in this study and the loss of some of the patients to follow up. We conducted careful sampling to include patients with different age, education, and FI disease status (see Additional file 1). The sampling strategy purposely also included some patients with low smartphone literacy or without a smartphone to not oversample high digital literacy patients or regular smartphone users. While this would likely only bias our baseline usability results in the direction of reduced usability (with low smartphone literacy users presumably giving lower scores), it also means that those patients, practically, could not participate in the follow-up assessment. At the same time, small-sample studies can be sufficient to conduct usability tests and can still reliably identify large effects or potential problems with an application [36]. As a further limitation, the verbally administered SUS questionnaires at the follow-up assessment should be mentioned. Although scores were comparable to the first assessment, we cannot fully rule out a potential bias introduced by the different modes of assessments.

## Conclusions

Based on the findings from this pilot study, we recommend the usage of the eDiary for the documentation of FI in clinical trials or clinical practice for patients who own and use a smartphone. Our study contributes to measuring the patient perspective in FI clinical trials. We found that patients were generally very open towards using their smartphones to document FI using our eDiary, which indicates the potential value of such systems. The system we tested, along with the screenshots and feedback from usability testing, may inspire future developments of similar systems for other diseases.

## Supplementary Information

**Additional file 1: Appendices 1 and 2.** eDiary screenshots and tables with additional patient chracteristics and self-reported stool problems.

**Additional file 2: Appendix 3.** Anonymized dataset used in the publication.

## Data Availability

The dataset supporting the conclusions of this article is included within the article (and its additional files).
