# Supplementary material for: Usability evaluation of the Computer-Based Health Evaluation System (CHES) eDiary for patients with faecal incontinence: a pilot study
Source: BMC Med Inform Decis Mak. 2022 Mar 28;22:81. doi: 10.1186/s12911-022-01818-5 (PMC8962247; doi:10.1186/s12911-022-01818-5)
Supplement: Supplementary file 1 — Additional file 1: Appendices 1 and 2. eDiary screenshots and tables with additional patient chracteristics and self-reported stool problems. [file 12911_2022_1818_MOESM1_ESM.docx]

# Appendices

##
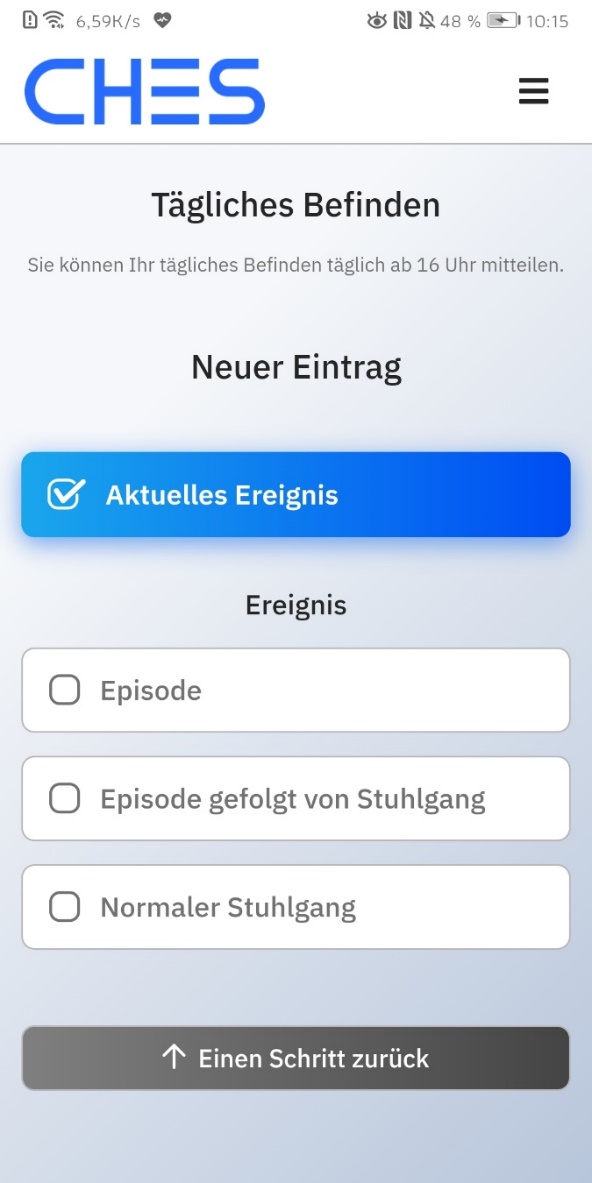

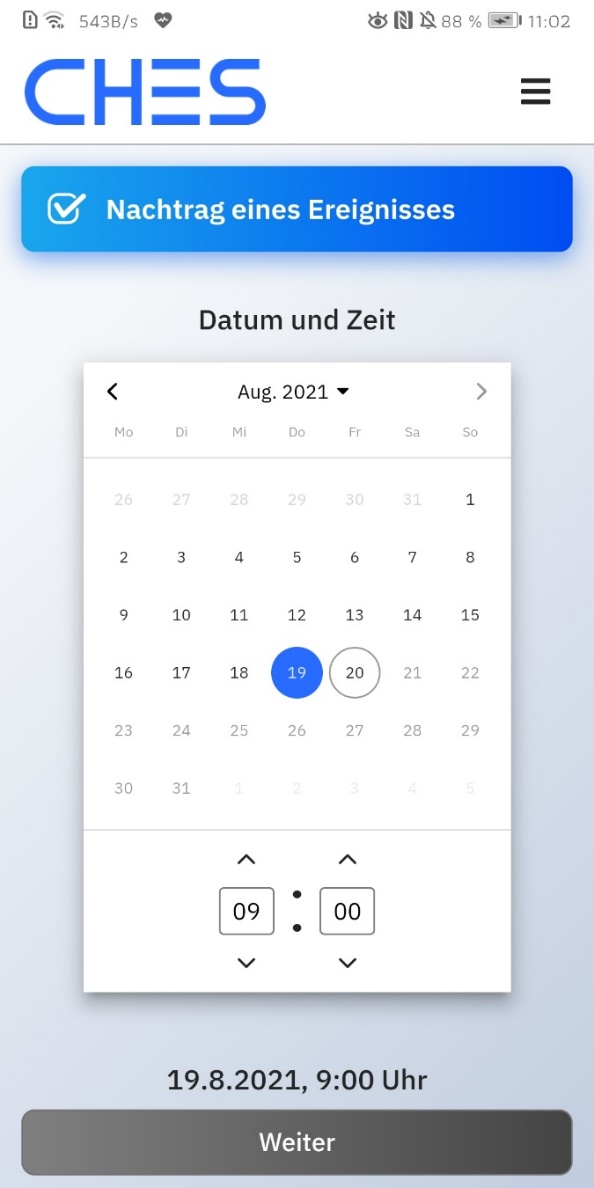
Appendix 1: eDiary Screenshots [original in German language, with translations]

Translations of text in the screenshots:

| Daily health status  You can enter your daily health status everyday past 4 pm.  New entry  Recent episode  Event  Episode  Episode followed by defecation  Normal defecation  Go back | Enter a past event  Date and time  [select a date]  Continue |
| --- | --- |


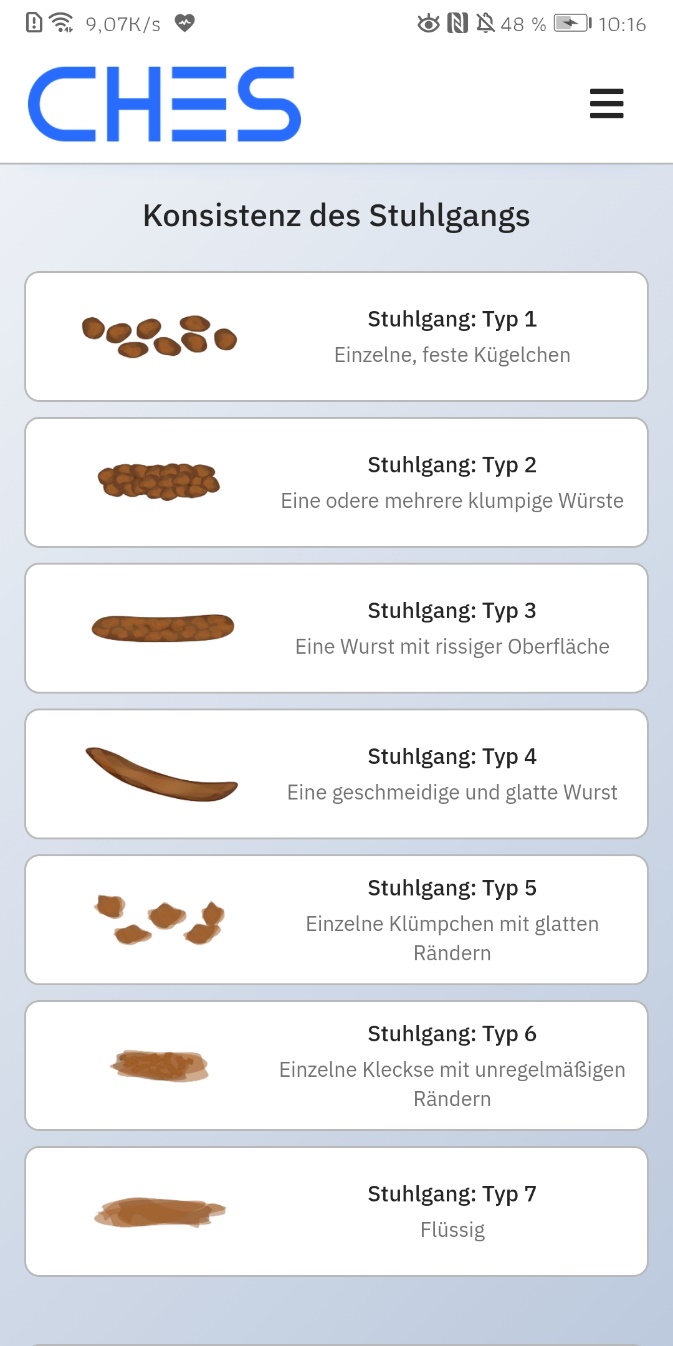

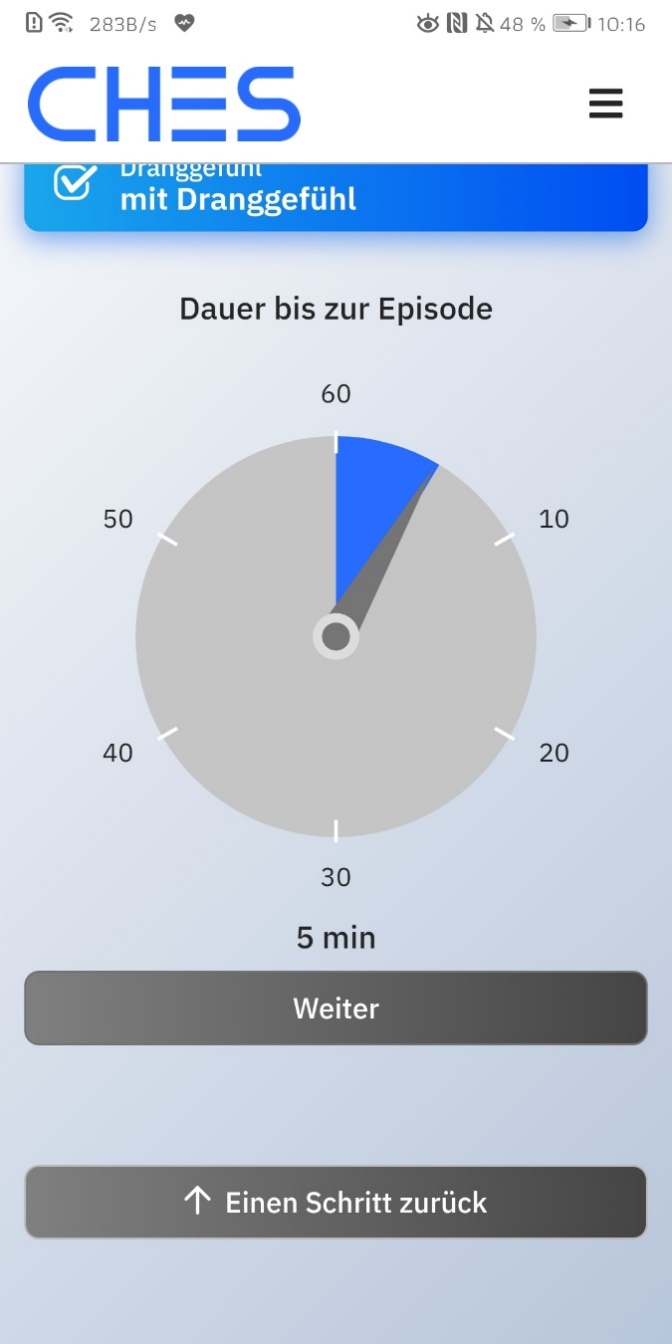


Translations of text in the screenshots:

| Time to episode  [Enter time]  Continue  Go back | Consistency of stool  Type 1: Separate, hard lumps  Type 2: Sausage shaped but lumpy  Type 3: Like a sausage, but with cracks  Type 4: Like a sausage or snake, smooth and soft  Type 5: Soft blobs with clear cut edges  Type 6: Fluffy pieces with mushed edges, a mushy stool  Type 7: Watery/Liquid |
| --- | --- |


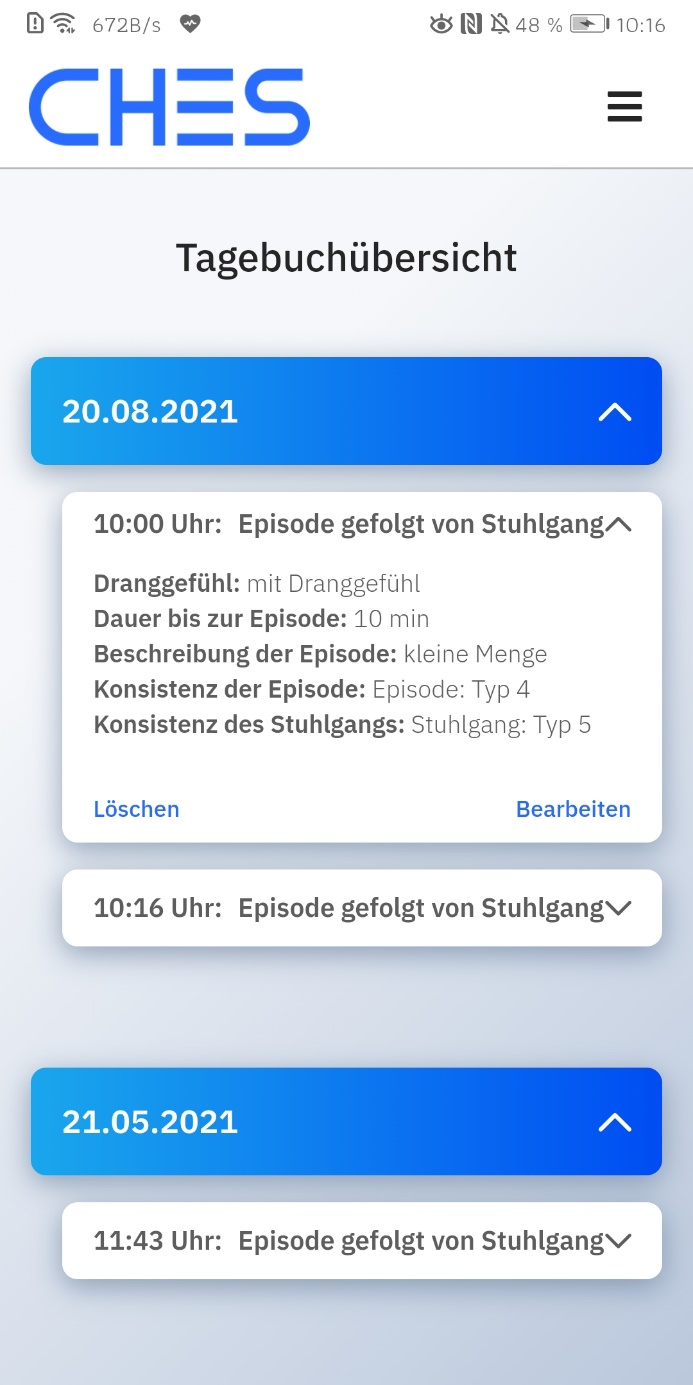


Translation of text in the screenshot:

| Overview  [date]  10:00 o’clock: Episode followed by defecation  Urge: with urge  Time to episode: 10 min.  Description of episode: small amount  Consistency of episode: Type 4  Consistency of stool: Type 5  Delete Edit  10:16: Episode followed by defacation  [date]  11:43: Episode followed by defecation |  |
| --- | --- |

## Appendix 2: Additional patient characteristics and self-reported stool problems

**Table A1. Patient characteristics**

| Characteristics | N = 14 |
| --- | --- |
| sex N (%) |  |
| male | 3 (21) |
| female | 11 (79) |
| age |  |
| mean | 67.4 |
| SD | 10.7 |
| education N (%) |  |
| compulsory school graduation (apprenticeship) | 10 (72) |
| matura (further education) | 3 (21) |
| university degree | 1 (7) |
| occupation N (%) |  |
| part-time | 1 (7) |
| full-time | 2 (14) |
| retired | 11 (79) |
| relationship status N (%) |  |
| married or long-term relationship (>1a) | 5 (36) |
| divorced/widowed | 8 (57) |
| single | 1 (7) |
| living situation N (%) |  |
| alone | 6 (43) |
| with partner/family/kids | 8 (57) |
| internet usage N (%) |  |
| confident in internet knowledge – yes | 9 (64) |
| confident in internet knowledge – no | 6 (36) |
| devices used to access the internet (multiple answers possible) N (%) |  |
| desktop PC | 3 (21) |
| laptop | 2 (14) |
| tablet | 3 (21) |
| smartphone | 10 (71) |
| frequency of internet usage N (%) |  |
| once per month | 1 (8) |
| one to three times per week | 2 (17) |
| once per day | 3 (25) |
| multiple times a day | 6 (50) |
| missing^a^ | 2 |

Note: ^a^ missing data were not included in the calculation of percentages.

**Table A2. Self-reported bowel and stool problems**

| Stool information and problems | N = 14 |
| --- | --- |
| stool consistency N (%) |  |
| soft | 2 (14) |
| varying | 6 (43) |
| solid | 6 (43) |
| missing^a^ | 2 |
| having to push hard during defecation N (%) |  |
| never | 9 (64) |
| sometimes (<1 per week) | 0 |
| frequently (>1 per week) | 3 (21) |
| most of the time (daily) | 2 (14) |
| flatulence N (%) |  |
| never | 2 (14) |
| sometimes (<1 per week) | 2 (14) |
| frequently (>1 per week) | 5 (36) |
| most of the time (daily) | 5 (36) |
| faecal incontinence (solid stool) N (%) |  |
| never | 7 (50) |
| sometimes (<1 per week) | 5 (36) |
| frequently (>1 per week) | 2 (14) |
| most of the time (daily) | 0 |
| faecal incontinence (watery stool) N (%) |  |
| never | 3 (21) |
| sometimes (<1 per week) | 3 (21) |
| frequently (>1 per week) | 5 (36) |
| most of the time (daily) | 3 (21) |
| feeling of inability to completely empty bowels N (%) |  |
| never | 3 (21) |
| sometimes (<1 per week) | 4 (29) |
| frequently (>1 per week) | 1 (7) |
| most of the time (daily) | 6 (43) |
| extent of trouble caused by bowel problems N (%) |  |
| none | 0 |
| a little | 2 (14) |
| quite a bit | 0 |
| very much | 12 (86) |

Note: ^a^ missing data were not included in the calculation of percentages.
